# Surface phytolith and pollen assemblages of a low-latitude subtropical region in Southwest China and their implications for vegetation and climate

**DOI:** 10.3389/fpls.2022.1007612

**Published:** 2022-10-04

**Authors:** Min Wang, Qing Yang, Wanshu Yang, Lin Shi, Yu Zhang, Zining Zhou, Wuqi Zhang, Hongbo Zheng

**Affiliations:** ^1^Yunnan Key Laboratory of Earth System Science, School of Earth Science, Yunnan University, Kunming, China; ^2^Yunnan Key Laboratory of Plateau Geographical Processes and Environmental Change, Faculty of Geography, Yunnan Normal University, Kunming, China; ^3^School of History and Archives Science, Yunnan University, Kunming, China; ^4^School of Earth and Environmental Sciences, The University of Queensland, Bribane, QLD, Australia

**Keywords:** subtropical region, modern vegetation, surface soil phytolith, surface soil pollen, climate

## Abstract

Phytoliths, as a newly developing plant proxy, have broad application prospects in paleoclimate and paleoethnobotany. However, the shortage of studies regarding tropical-subtropical plants and topsoil phytoliths interferes with the research progress on primitive humanity’s utilization of plant resources and paleoclimate in the region. This research focuses on the subtropical mountainous region with a monsoon climate of low latitudes in Southwest China to conduct phytolith morphology analysis of living plants and phytolith/pollen assemblages of topsoil to reveal the indicative significance of vegetation and climate. A total of 111 species from 50 families, including 73 species from 33 tree/shrub families, 31 species from 12 herb families and 7 species from 5 fern families, were collected for morphological characteristics analysis, as well as 19 topsoil specimens for phytolith and pollen assemblage analysis. The results suggest that phytoliths are mainly deposited *in situ*, with assemblages of topsoil corresponding well with plant types in the quadrat and being able to exhibit constructive species in small regions. In comparison, pollen assemblages of topsoil dominantly respond to regional vegetation due to their long-distance transportation and widespread presence, in addition to their characteristics that correspond to the vegetation in the quadrat. The topsoil phytolith assemblages are mainly based on the elongate-bulliform flabellate-square/rectangle-broadleaf-types (including spheroid echinate), and the vegetation types indicate the subtropical climate. In addition, phytolith assemblages of Poaceae are dominated by collapsed saddle-bulliform flabellate square/rectangle-elongate-point, reflecting warm and humid conditions. The pollen assemblages mainly consist of *Pinus*, *Betula*, *Alnus*, deciduous *Quercus*, Euphorbiaceae, Rhamnaceae and *Polygonum*, reflecting tropical-subtropical plant communities and indicating warm and humid conditions. Overall, phytolith and pollen assemblages have unique characteristics and are thus explicitly representative of the low-latitude subtropical monsoon climate.

## Introduction

As good biological proxies, pollen and phytoliths have attracted extensive attention from paleoclimate scholars due to their sensitivity to environmental changes as well as their abundance and wide distribution (Herzschuh et al., 2010; Stebich et al., 2015; Lu et al., 2018). At present, studies on the reconstruction of past climate changes based on pollen and phytoliths have been carried out in various regions of the world, making important contributions to paleoclimate studies (Whitmore et al., 2005; Shen et al., 2006; Lu et al., 2007).

Southwest China is a key area to study the history of the southwest monsoon. In recent years, studies on the reconstruction of paleovegetation, paleoclimate, and paleomonsoon in Southwest China based on fossil pollen have been increasing gradually (Xiao et al., 2014; Zhang et al., 2020). Nevertheless, studies on topsoil pollen in China are mainly concentrated in northern China (Xu Q. H. et al., 2005, Lu et al., 2006; Xu et al., 2007; Li et al., 2008, 2012; Luo et al., 2010) and the Tibetan Plateau (Lu et al., 2004; Wei et al., 2018; Zhang et al., 2018; Qin, 2021), yet these studies conducted in southern China are relatively weak. Previous studies mainly rely on subjective empirical inference when interpreting the environmental significance of sedimentary pollen and phytoliths, failing to give accurate quantitative results. There are differences in types and contents between topsoil pollen/phytolith and pollen/phytolith assemblages from plant communities; moreover, phytoliths of different plants or different phytoliths of the same plant have distinct vegetation representativeness (Xu D. K. et al., 2005; Liu et al., 2020). This will affect the accuracy of paleoclimate reconstruction based on topsoil pollen and phytoliths. The vegetation representativeness level of topsoil pollen and phytoliths, therefore, is the critical issue restricting the accuracy of palaeovegetation and palaeoclimate restoration.

Phytoliths also play an irreplaceable role in interpreting the origins and domestication of prehistoric human use of plant resources and cultivated crops (Lu et al., 2007, 2009; Yang et al., 2013; Liu, 2016). Currently, studies regarding phytoliths of modern plants mainly focus on Poaceae (Li et al., 2005; Piperno, 2006; Huan et al., 2015). The silicon morphology of economic crops has also been widely studied, such as *Musa basjoo* (Ball et al., 2006; Horrocks et al., 2009), Palmae (Xu D. K. et al., 2005), *Cucurbita moschata* (Piperno et al., 2000), *Lagenaria siceraria* (Piperno and Stothert, 2003), *Manihot esculenta*, *Maranta arundinacea* and other tubers (Chandler-Ezell et al., 2006). However, the number of studies on phytoliths of Xylophyta is still relatively low. The possible reason for this lack of study is that the phytoliths of xylophyta vary greatly in morphology, and only a few phytoliths have taxonomic significance (Wang and Lu, 1993). If more detailed taxonomic identification of the phytoliths in xylophyta living in different habitats is conducted, phytoliths may become more accurate environmental indicators, similar to pollen’s implications for vegetation.

In recent years, scholars have intensified the research on the phytolith classification of xylophyta, such as Palmae (Xu D. K. et al., 2005; Fenwick et al., 2011), tropical xylophyta (Bai et al., 2020), common xylophyta in China (Ge, 2016) and xylophyta from Mozambique, East Africa (Mercader et al., 2009). Although there has been gains in understanding, there is a wide variety of species of xylophyta, especially in tropical-subtropical regions, which need to be supplemented by further information on their phytoliths. Compared with pollen, phytoliths have an irreplaceable advantage in local vegetation restoration. Systematic research on the phytolith classification of xylophyta can provide a fundamental reference to vegetation zones, plant communities, and even typical species for pass human activity studies (An et al., 2015).

Some advances have been made in the study of topsoil phytoliths in revealing climatic environments. Wang and Lu (1993) qualitatively summarized cold and warm typal phytoliths according to the spatial distribution of different phytolith types in Chinese topsoil. Since the characteristics of phytolith assemblages on the surface of peatland in northeast China are significantly correlated with latitude, altitude, and humidity, paleoclimate can be inferred by the climate transfer function (Zhang et al., 2008). The study on phytolith assemblage characteristics in Changbai Mountain found that the paleoenvironmental pattern deduced from the phytolith assemblage was consistent with the results of pollen analysis (Li et al., 2011). However, overall, research on topsoil phytoliths in China is inadequate, particularly in tropical-subtropical regions.

In this paper, the semihumid evergreen broad-leaved forest area of Hengduan Mountain, Southwest China, was selected as the area of interest, and modern plants from Nongke Mountain, Cangyuan, were collected for phytolith morphology analysis; topsoil pollen and phytolith studies in the study area were also conducted. This study is a useful supplement to the phytoliths of modern plants, topsoil phytoliths and pollen in Southwest China, providing a reliable reference and basis for the accurate interpretation and quantitative reconstruction of fossil pollen in the study area.

## Location and vegetation information

**Figure 1:**
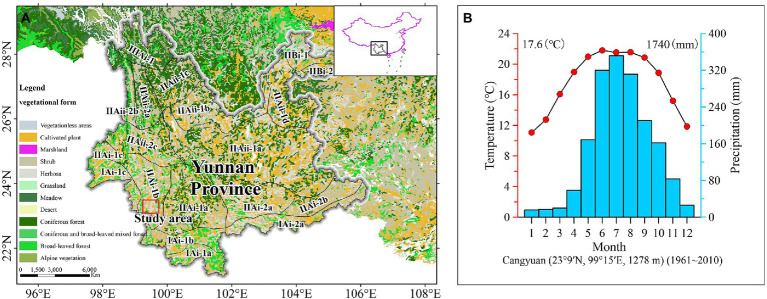
Vegetation map of regional location **(A)** and mean annual temperature and annual precipitation in the research area **(B)**.

The study area is located in the middle part of the Lancang River Basin with complex terrain and a great diversity of habitat in western Yunnan, China. Affected by the Indian Monsoon, the monsoon climate of low latitudes in subtropical mountains was warmer and damper (Figure 1), with a mean annual temperature of 17.6°C and annual precipitation of 1740 mm.^1^ The area is part of the semihumid evergreen broad-leaved forest region in the Hengduan Mountains and largely overlaps with the bamboo-rich area in Asia. The vegetation species are abundant in this area, containing various broad-leaved woody plants (Yunnan Vegetation Compilation Group, 1986), mainly *Castanopsis hystrix*, *Castanopsis indica*, and *Castanopsis ferox*. *Pinus yunnanensis* forest is widely distributed in the region, while *Pinus kesiya* is sporadically distributed. Vegetation such as *Schima reinw*, *Alnus nepalensis*, *Quercus acutissima* and *Quercus variabilis* are often scattered on the grassy slopes of barren hills (Yunnan Vegetation Compilation Group, 1986). According to the sampling survey, 1,000–1,300 m in this region was mainly warm bamboo forest and warm evergreen-deciduous broad-leaved forest (The Bambusoideae, Fagaceae, Fabaceae, Moraceae are the main families. And also include Oleaceae, Betulaceae, Urticaceae and vine. Under the forest, Poaceae, *Trevesia palmata*, ferns are grown). 1,300–1950 m, there are mainly bamboo forest, pine forest, *quercus* forest and hazel forest in this region. Contains Euphorbiaceae, Betulaceae, Moraceae, Myricaceae, Juglandaceae, Taxodiaceae and Rhamnaceae. Poaceae, Zingiberaceae, Asteraceae and ferns grow under the forest. Corn land distribution in the region. 1,950–2,100 m, mainly pine forests and coniferous forests, including some evergreen broad-leaved components (Theaceae, Betulaceae, Rosaceae and Betulaceae. And Liliaceae, Asteraceae and Zingiberaceae grow under the forest).

## Materials and methods

### Plant and topsoil sampling

**Figure 2:**
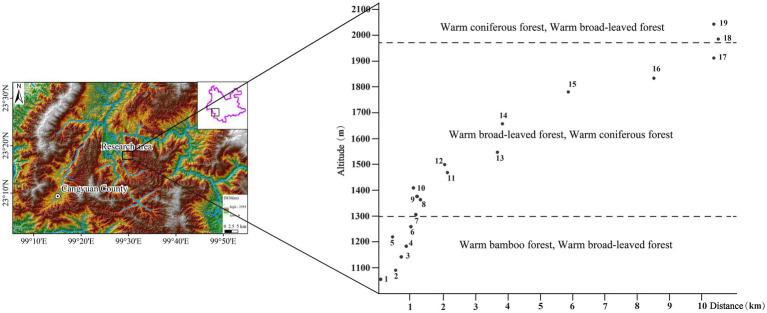
Geographical location of topsoil samples and vertical distribution of sampling points.

A total of 19 representative quadrats from different altitudes (covering the local mountain vertical vegetation belt) were selected in southern Hengduan Mountain (23°17.71′ ~ 23°20.26′N, 99°28.69′ ~ 99°34.97′E, altitude: 1,050 ~ 2,100 m) for plant and topsoil sampling in December 2020 in Cangyuan County. There were 111 species of plants, 19 topsoil samples and 1 moss sample (CYM10, as a parallel sample of CY10) collected (Figure 2).

Topsoil sample collection: A 1 m^2^ quadrat was selected, and topsoil samples were mixed of subsamples of topsoil from the top 2 cm from the 4 corners and the center point of the square quadrat by removing the turf. A total of 19 topsoil samples were used and covered the main vegetation types of the local mountain vertical vegetation zone, and the sampling point information and vegetation types are shown in Tables 1,2.

**Table 1:** Plant samples used in the experiment.

Number	Family	Genus	Species	Sampling site
1	Fagaceae		(1 type, not identified)	Cangyuan
2	Fagaceae	*Quercus*	*Quercus guyavifolia*	Cangyuan
3	Fagaceae	*Quercus*	*Quercus acutissima*	Cangyuan
4	Fagaceae	*Quercus*	*Quercus aliena*	Xishan, Kunming City
5	Fagaceae	*Quercus*	*Quercus variabilis*	Kunming botanical garden
6	Fagaceae	*Quercus*	*Quercus serrata*	Kunming botanical garden
7	Fagaceae	*Lithocarpus*	*Lithocarpus mairei*	Cangyuan
8	Fagaceae	*Lithocarpus*	*Lithocarpus glaber*	Kunming botanical garden
9	Fagaceae	*Cyclobalanopsis*	*Cyclobalanopsis glaucoides*	Kunming botanical garden
10	Fabaceae	*Erythrina*	*Erythrina stricta*	Cangyuan
11	Fabaceae	*Dalbergia*	*Dalbergia sissoo*	Cangyuan
12	Fabaceae	*Campylotropis*	*Campylotropis macrocarpa*	Cangyuan
13	Fabaceae	*Campylotropis*	*Campylotropis delavayi*	Cangyuan
14	Fabaceae	*Uraria*	*Uraria crinita*	Cangyuan
15	Fabaceae	*Bauhinia*	(1 type, not identified)	Cangyuan
16	Fabaceae	*Bauhinia*	*Bauhinia purpurea*	Cangyuan
17	Fabaceae	*Lespedeza*	*Lespedeza cuneata*	Cangyuan
18	Fabaceae	*Albizia*	*Albizia odoratissima*	Cangyuan
19	Moraceae		(2 type, not identified)	Cangyuan
20	Moraceae	*Ficus*	(4 type, not identified)	Cangyuan
21	Moraceae	*Ficus*	*Ficus maclellandii*	Cangyuan
22	Moraceae	*Broussonetia*	(1 type, not identified)	Cangyuan
23	Moraceae	*Maclura*	(1 type, not identified)	Kunming botanical garden
24	Lauraceae		(3 type, not identified)	Cangyuan
25	Lauraceae	*Cinnamomum*	*Cinnamomum pittosporoides*	Cangyuan
26	Lauraceae	*Neocinnamomum*	*Neocinnamomum caudatum*	Cangyuan
27	Lauraceae	*Machilus*	*Machilus nanmu*	Xishan, Kunming City
28	Celastraceae	*Celastrus*	(1 type, not identified)	Cangyuan
29	Celastraceae	*Celastrus*	*Celastrus paniculatus*	Cangyuan
30	Celastraceae		(1 type, not identified)	Cangyuan
31	Acanthaceae		(3 type, not identified)	Cangyuan
32	Anacardiaceae	*Rhus*	*Rhus chinensis*	Cangyuan
33	Euphorbiaceae		(2 type, not identified)	Cangyuan
34	Euphorbiaceae	*Phyllanthus*	*Phyllanthus emblica*	Cangyuan
35	Alangiaceae	*Alangium*	*Alangium chinense*	Cangyuan
36	Primulaceae	*Embelia*	*Embelia ribes*	Cangyuan
37	Aquifoliaceae	*Ilex*	(1 type, not identified)	Cangyuan
38	Ericaceae	*Gaultheria*	*Gaultheria leucocarpa*	Cangyuan
39	Ericaceae		(1 type, not identified)	Xishan, Kunming City
40	Betulaceae	*Alnus*	*Alnus nepalensis*	Cangyuan
41	Betulaceae	*Alnus*	*Alnus cremastogyne*	Cangyuan
42	Juglandaceae	*Juglans*	*Juglans regia*	Cangyuan
43	Apocynaceae	*Epigynum*	*Epigynum auritum*	Cangyuan
44	Apocynaceae	*Aganosma*	*Aganosma cymosa*	Cangyuan
45	Palmae	*Phoenix*	*Phoenix dactylifera*	Cangyuan
46	Palmae		(1 type, not identified)	Cangyuan
47	Meliaceae	*Cipadessa*	*Cipadessa baccifera*	Cangyuan
48	Verbenaceae	*Vitex*	(1 type, not identified)	Cangyuan
49	Rosaceae	*Pygeum*	*Pygeum topengii*	Cangyuan
50	Rosaceae	*Dichotomanthes*	*Dichotomanthes tristaniicarpa*	Cangyuan
51	Loranthaceae	*Dendrophthoe*	(1 type, not identified)	Cangyuan
52	Salicaceae	*Flacourtia*	*Flacourtia rukam*	Cangyuan
53	Theaceae	*Schima*	(1 type, not identified)	Cangyuan
54	Theaceae	*Schima*	*Schima superba*	Cangyuan
55	Theaceae	*Camellia*	*Camellia sinensis*	Cangyuan
56	Theaceae	*Anneslea*	*Anneslea fragrans*	Cangyuan
57	Theaceae	*Eurya*	*Eurya alata*	Cangyuan
58	Rhamnaceae	*Rhamnus*	*Rhamnus leptophylla*	Cangyuan
59	Rhamnaceae	*Hovenia*	*Hovenia acerba*	Kunming botanical garden
60	Santalaceae	*Osyris*	*Osyris quadripartita*	Cangyuan
61	Araliaceae	*Trevesia*	*Trevesia palmata*	Cangyuan
62	Urticaceae		(1 type, not identified)	Cangyuan
63	Urticaceae	*Oreocnide*	*Oreocnide frutescens*	Cangyuan
64	Melastomataceae	*Melastoma*	*Melastoma malabathricum*	Cangyuan
65	Primulaceae	*Myrsine*	*Myrsine africana*	Cangyuan
66	Bignoniaceae	*Oroxylum*	*Oroxylum indicum*	Cangyuan
67	Magnoliaceae		(1 type, not identified)	Cangyuan
68	Magnoliaceae	Michelia	*Michelia figo*	Xishan, Kunming City
69	Dioscoreaceae	*Dioscorea*	*Dioscorea hispida*	Cangyuan
70	Dioscoreaceae	*Dioscorea*	*Dioscorea alata*	Cangyuan
71	Cupressaceae	*Cunninghamia*	*Cunninghamia lanceolata*	Cangyuan
72	Pinaceae	*Pinus*	*Pinus yunnanensis*	Cangyuan
73	Pinaceae	*Pinus*	*Keteleeria evelyniana*	Xishan, Kunming City
74	Poaceae	*Arundinella*	(1 type, not identified)	Cangyuan
75	Poaceae	*Imperata*	*Imperata cylindrica*	Cangyuan
76	Poaceae	*Phacelurus*	*Phacelurus latifolius*	Cangyuan
77	Poaceae	*Cymbopogon*	*Cymbopogon citratus*	Cangyuan
78	Poaceae	*Dendrocalamus*	*Dendrocalamus peculiaris*	Cangyuan
79	Poaceae	*Bambusa*	*Bambusa beecheyana*	Cangyuan
80	Poaceae	*Capillipedium*	*Capillipedium parviflorum*	Cangyuan
81	Poaceae	*Dendrocalamus*	*Dendrocalamus giganteus*	Cangyuan
82	Poaceae	*Heteropogon*	*Heteropogon contortus*	Cangyuan
83	Poaceae	*Arthraxon*	*Arthraxon lancifolius*	Cangyuan
84	Poaceae	*Cymbopogon*	*Cymbopogon goeringii*	Cangyuan
85	Poaceae	*Oplismenus*	*Oplismenus undulatifolius*	Cangyuan
86	Poaceae	*Eleusine*	*Eleusine indica*	Cangyuan
87	Poaceae	*Setaria*	*Setaria pumila*	Cangyuan
88	Asparagaceae	*Ophiopogon*	*Ophiopogon mairei*	Cangyuan
89	Asparagaceae	*Polygonatum*	(1 type, not identified)	Cangyuan
90	Araceae	*Alocasia*	(1 type, not identified)	Cangyuan
91	Lamiaceae		(1 type, not identified)	Cangyuan
92	Lamiaceae	*Elsholtzia*	*Elsholtzia rugulosa*	Cangyuan
93	Zingiberaceae	*Kaempferia*	(1 type, not identified)	Cangyuan
94	Zingiberaceae		(1 type, not identified)	Cangyuan
95	Asteraceae	*Artemisia*	*Artemisia argyi*	Cangyuan
96	Asteraceae	*Bidens*	*Bidens pilosa*	Cangyuan
97	Asteraceae	*Vernonia*	*Vernonia esculenta*	Cangyuan
98	Asteraceae	*Duhaldea*	*Duhaldea cappa*	Cangyuan
99	Polygonaceae	*Polygonum*	*Polygonum chinense*	Cangyuan
100	Musaceae	*Musa*	*Musa basjoo*	Cangyuan
101	Gesneriaceae		(1 type, not identified)	Cangyuan
102	Orchidaceae	*Cleisostoma*	*Cleisostoma paniculatum*	Cangyuan
103	Berberidaceae	*Dysosma*	*Dysosma majoensis*	Cangyuan
104	Equisetaceae	*Equisetum*	*Equisetum ramosissimum*	Cangyuan
105	Pteridaceae	*Pteris*	(1 type, not identified)	Cangyuan
106	Pteridaceae	*Pteris*	*Pteris cretica*	Cangyuan
107	Polypodiaceae	*Drynaria*	(1 type, not identified)	Cangyuan
108	Polypodiaceae	*Pyrrosia*	(1 type, not identified)	Cangyuan
109	Athyriaceae	*Athyrium*	(1 type, not identified)	Cangyuan
110	Ophioglossaceae	*Botrychium*	*Botrychium ternatum*	Cangyuan
111	Dennstaedtiaceae	*Pteridium*	*Pteridium aquilinum*	Cangyuan

**Table 2:** Original records of surface soil sample collection near Nongke Village, Meng Province Town, Cangyuan County.

Sample points	Latitude (N)	Longitude (E)	Elevation (m)	Plant community
Points 1	23°18.0589′	99°28.6919′	1,059	Trees are mainly Fabaceae and Moraceae, including Oleaceae, *Pistacia weinmanniifolia*, *Bauhinia purpurea*, *Cipadessa baccifera*, and understory Acanthaceae.
Points 2	23°18.1102′	99°29.0518′	1,090	The trees are mainly Fabaceae and Moraceae. There are many vines in the forest. There are Poaceae, *Trevesia palmata*, a small amount of ferns and *Kalopanax septemlobus* under the forest.
Points 3	23°18.104′	99°29.2903′	1,148	Evergreen, deciduous broad-leaved forest, mixed with a variety of trees containing Betulaceae plants, dominated by Urticaceae, shrubs containing Fabaceae, Urticaceae.
Points 4	23°18.0664′	99°29.4343′	1,181	Wild bamboo forest, dominated by Celastraceae.
Points 5	23°17.9572′	99°29.0148′	1,227	Tree communities dominated by *Quercus* in Fagaceae, including Fabaceae, Euphorbiaceae, Loranthaceae, Celastraceae, and Poaceae undergrowth.
Points 6	23°18.0773′	99°29.5373′	1,265	Fabaceae, Acanthaceae, belonging to deciduous forest, shrubbery.
Points 7	23°17.9209′	99°29.6289′	1,308	Mainly bamboo forest.
Points 8	23°17.7958′	99°29.6772′	1,366	Trees are dominated by *Pinus*, with Poaceae and *Ageratina adenophora* growing under the forest (Zea mays fields are seen near the sampling points).
Points 9	23°17.793′	99°29.622′	1,375	Trees are dominated by Fagaceae, and the forest contains Moraceae and Myrsinaceae. A large number of vine plants are grown under the forest (Zea mays fields are seen near the sampling points).
Points 10	23°17.746′	99°29.5848′	1,409	The low-lying *Quercus*, belonging to arbor and shrub, contains Euphorbiaceae, Celastraceae, Fabaceae, and Rhamnaceae plants. Zingiberaceae grows under the forest, and the surface is mossy (mainly).
Points 11	23°19.8485′	99°29.7853′	1,449	*Quercus* forest, shrub Fabaceae; Poaceae are the main families under the forest, which can be found in *Phoenix loureiroi* and *Artemisia*.
Points 12	23°20.256′	99°29.7516′	1,500	Trees include *Pinus*, *Quercus*, *Broussonetia papyrifera*, *Juglans regia*, *Zanthoxylum*, *Phyllanthus emblica*, Fabaceae and unknown species. Under the forest, the dominant species were *Phoenix loureiroi* and Poaceae.
Points 13	23°19.728′	99°30.7019′	1,551	The genus *Quercus* of Fagaceae was the main species, and *Pistacia weinmanniifolia* of Fabaceae was the most common species. There were Ficus of Moraceae, Lauraceae, and *Phoenix loureiroi* under the forest Poaceae, *Alpinia japonica* (suspected as artificial cultivation), *Artemisia argyi*, *Ageratina adenophora* and Labiatae were found.
Points 14	23°18.9245′	99°30.9243′	1,659	Pine forest (include *Ficus microcarpa*), mainly Fabaceae *APistacia weinmanniifolia*, containing Rhamnaceae, Poaceae and *Artemisia argyi* under the forest.
Points 15	23°18.0171′	99°32.1412′	1,789	Pinus forest, see Fagaceae plant deciduous *Quercus*, evergreen *Quercus*, more *Cunninghamia lanceolata*, see Poaceae (mainly), *Ageratina adenophora*, *Pogonatherum crinitum*.
Points 16	23°17.4435′	99°33.8201′	1,839	For evergreen deciduous broad-leaved forest, Betulaceae deciduous trees and Theaceae *Schima*, including Lauraceae.
Points 17	23°17.8703′	99°34.9011′	1,902	The trees have hazelnut forest, see *Eurya japonica*, and there are Fabaceae, Theaceae, and Bambusoideae in the forest; shrubs have *Paeonia delavayi*, D*uhaldea cappa*, *Ageratina adenophora* and ferns.
Points 18	23°17.7105′	99°34.9675′	1,981	There are many woody plants, such as Theaceae, Betulaceae, *Rhus chinensis*, *Pygeum topengii* and so on. Most of them are *Ageratina adenophora* and Labiatae. The pine forest is evergreen tree.
Points 19	23°19.0789′	99°34.9060′	2,031	Vegetation is mainly pine forest, coniferous forest, including *Alnus*, undergrowth Liliaceae, Asteraceae, Zingiberaceae.

Plant sample collection: whole plants of herbaceous plants were collected, including roots, stems, leaves and spikes. Mature leaves were mainly collected from woody plants, with only a small number of fine branches also collected. All plant specimen samples were encapsulated individually in cowhide envelopes. In addition, a small number of samples for the analysis of modern plant phytoliths were collected in April and September 2021 in Xishan, Kunming and Kunming Botanical Garden, respectively.

### Identification of plant species

Species identification was performed on all collected plant samples. First and foremost, a few common species of plant samples were identified by a botany instructor at Yunnan University, while most of the samples were delivered to Tsingke Biotechnology Co., Ltd. to determine genus and species by DNA barcoding molecular identification technology, usually identified to the genus level, some plants identified to family. The taxonomic information and Latin names of the plant species used are collated with reference to http://www.iplant.cn (Table 1).

### Pretreatment methods of plant phytoliths, topsoil phytoliths, and pollen

Plant phytolith extraction: every part of each specimen was cleaned with distilled water in an ultrasonic water bath to remove adhering particles and then dried in an air drying box. The dried materials were cut into smaller parts and placed in separate tubes, and 20 ml (or enough to submerge the materials) saturated nitric acid was added to each tube and left for one night. The next day, the tubes with materials were heated in a water bath until the solution became clear and transparent. After removing the supernatant, the solutions were centrifuged and rinsed with distilled water 3 times and then with ethyl alcohol twice. Then, the extracted phytoliths in each tube were mounted on separate slides using Canada Balsam for further observation.

Topsoil phytolith extraction: A subsample of 3 g was taken from each collected dried topsoil sample. The dried materials were sequentially processed as follows: (1) organics were removed with hydrogen peroxide; (2) carbonates were dissolved with 10% hydrochloric acid; (3) flotation of phytoliths was accomplished using a ZnBr_2_ solution (density 2.35 g/cm^3^); and (4) after cleaning the flotation sample with distilled water and ethyl alcohol, each subsample was mounted on separate slides using Canada Balsam for further observation.

Pollen extraction: A subsample of 3 g was taken from each collected dried topsoil sample. The dried materials were sequentially processed as follows: (1) 10% hydrochloric acid was added; (2) 10% potassium hydroxide was added and heated in a water bath to remove organics; (3) 40% hydrofluoric acid was added to the cleaned sample and heated in a water bath to remove the silicate; (4) 15% hydrochloric acid was added and heated in a water bath to remove the soluble fluoride from the sample; (5) the samples were dehydrated by adding glacial acetic acid, and (6) after removing the supernatant, the solutions were added to a mixture of 9:1 acetic anhydride and concentrated sulfuric acid and heated in a water bath for 5 min. After passing through a 7 μm sieve, each subsample was mounted on separate slides adding glycerol for observation.

### Identification and statistics of phytoliths and pollen samples

The average number of phytoliths and pollen in topsoil samples was over 400 grains per sample, where the percentage of phytoliths was calculated excluding two types, stomata and sponge spicules; the percentage of terrestrial pollen was calculated based on the sum of shrubs and terrestrial herbs, while the percentage of pollen was calculated based on the percentage of pollen of trees, shrubs, terrestrial herbs and ferns. Finally, cluster analysis was performed using Tilia and generating phytolith and pollen percentage maps (Grimm, 2004).

## Results

### Phytolith morphology analysis of modern plants

The results of the field vegetation survey and sampling suggest that the vegetation types of south Hengduan Mountain mainly consist of Fabaceae, Euphorbiaceae, Celastraceae, Fagaceae, Moraceae, Lauraceae, Theaceae, Palmae, Poaceae, Asteraceae and ferns. Fagaceae, Fabaceae and Moraceae account for a relatively large proportion in forests. The vegetation composition of the sampling area changed slightly with elevation. A total of 111 genera and 50 families of modern plant samples were collected, including 73 genera and 33 families of trees and shrubs, 31 genera and 12 families of herbs, and 7 genera and 5 families of ferns. The morphological characteristics of the phytoliths of each family and genus are as follows.

(1) Phytolith morphology of trees and shrubs.

**PLATE 1:**
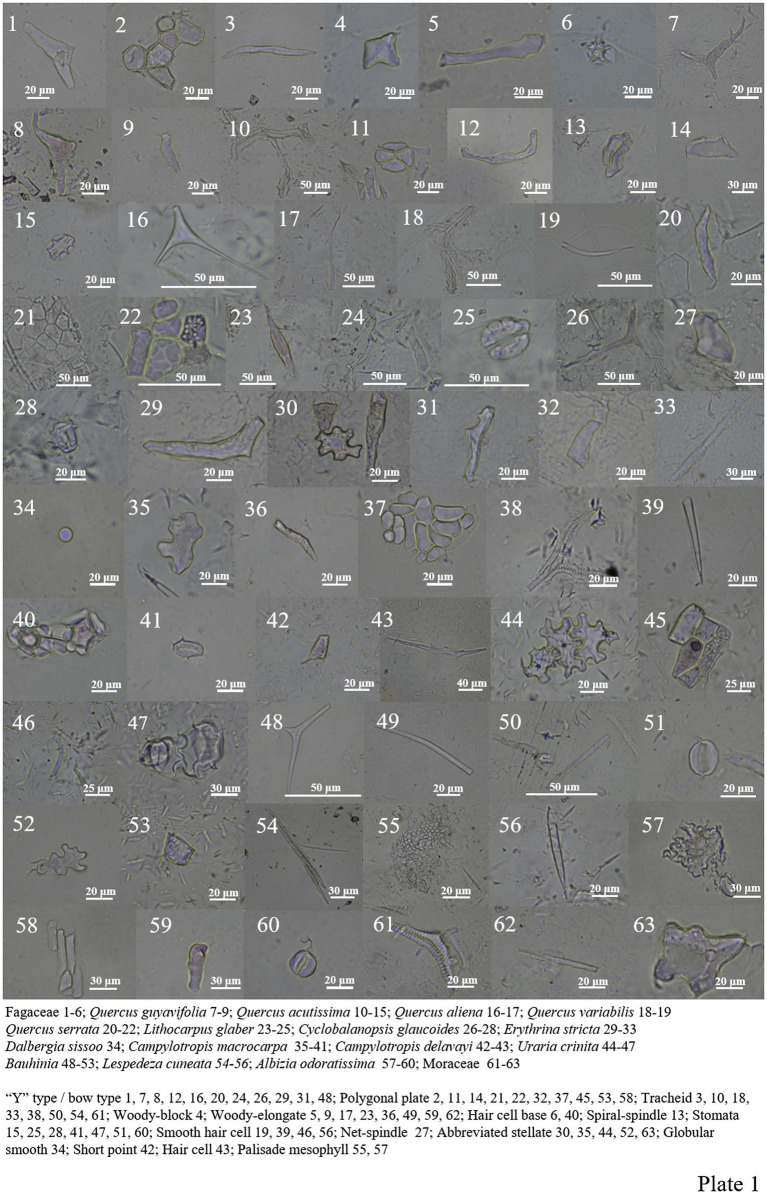


Among the 9 Fagaceae species analyzed, “Y” type/bow type, spiral-spindle, net-spindle, woody-elongate/block, tracheid, polygonal plate, hair cell, hair cell base and silicified stomate (Plate 1).

A total of 12 different types of phytoliths were produced in 13 Fabaceae plants (Plate 1, 29–60). “Y” type/bow type, abbreviated stellate, polygonal plate, globular smooth, short point, tracheid, woody elongated, epidermal cell, smooth hair cell, hair cell base, palisade mesophyll and siliconized stomatas. Many silicified stomata were found in *Bauhinia,* and a large number of smooth hair cells were found in *Campylotropis macrocarpa* (Plate 1, 35–41).

**PLATE 2:**
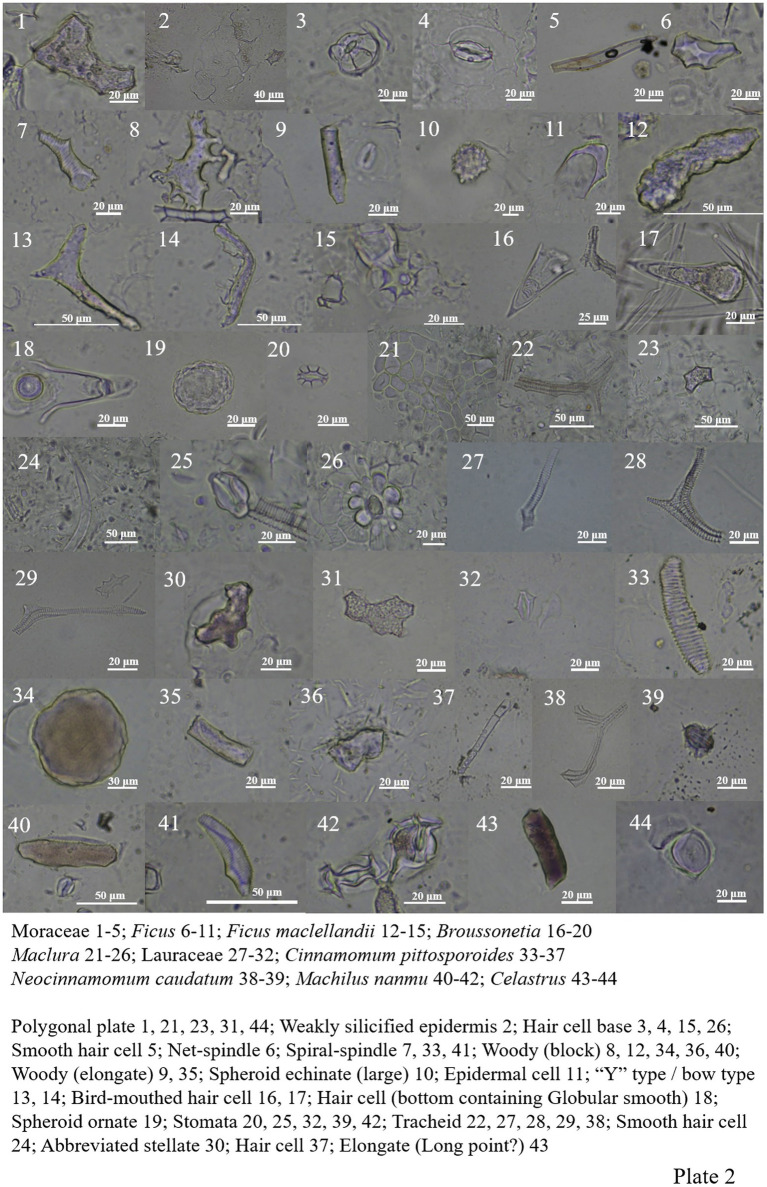


Moraceae contains most of the Fagaceae and Fabaceae types, and spheroid echinate (large), spheroid ornate and bird-mouthed hair cell were also seen (Plate 1, 61–63; Plate 2, 1–26).

Lauraceae contains most of the Fagaceae types (except net-spindle and abbreviated stellate (Plate 2, 27–39), and more siliconized stomatas are found in *Machilus nanmu* (Plate 2, 40–42).

**PLATE 3:**
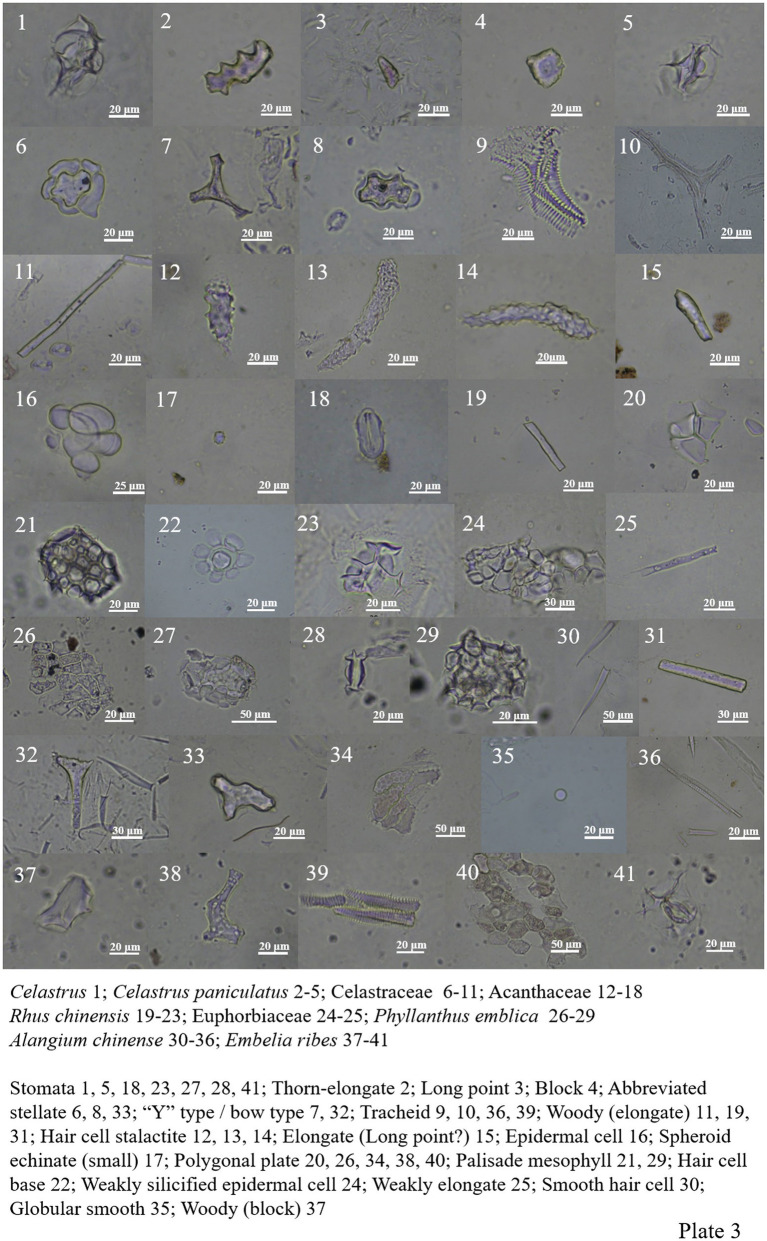


The morphology of phytoliths in Celastraceae includes woody-elongate, thorn-elongate, long point, block type, ‘Y’ type/bow type, abbreviated stellate, polygonal plate, tracheid, and siliconized stomata (Plate 2, 43–44; Plate 3, 1–11).

Acanthaceae includes elongate, epidermal cell, spheroid echinate (small), hair cell stalactite and silicified stomata, among which hair cell stalactite accounts for the majority of phytolith types (Plate 3, 12–18).

Anacardiaceae contains a large number of epidermal cells (Kealhofer and Piperno, 1998). *Rhus chinensis* (Plate 3, 19–23) includes not only a large number of polygonal plates but also woody-elongate, palisade mesophyll, hair cell base, and silicified stomata.

Phytoliths are also abundant in Euphorbiaceae (Alexandre et al., 2012). The results show that Euphorbiaceae contains polygonal plate, woody elongate, weakly silicified epidermis, silicified stomata and silicified mesophyll tissue (honeycomb type; Plate 3, 24–29).

*Alangium chinense* in Alangiaceae contains abundant phytoliths, including the “Y” type/bow type, abbreviated stellate, polygonal plate, tracheid, woody-elongate, globular smooth, and a large number of smooth hair cells (Plate 3, 30–36).

*Embelia ribes* in Primulaceae include the woody block, tracheid, silicified stomata, and numerous polygonal plates (Plate 3, 37–41).

**PLATE 4:**
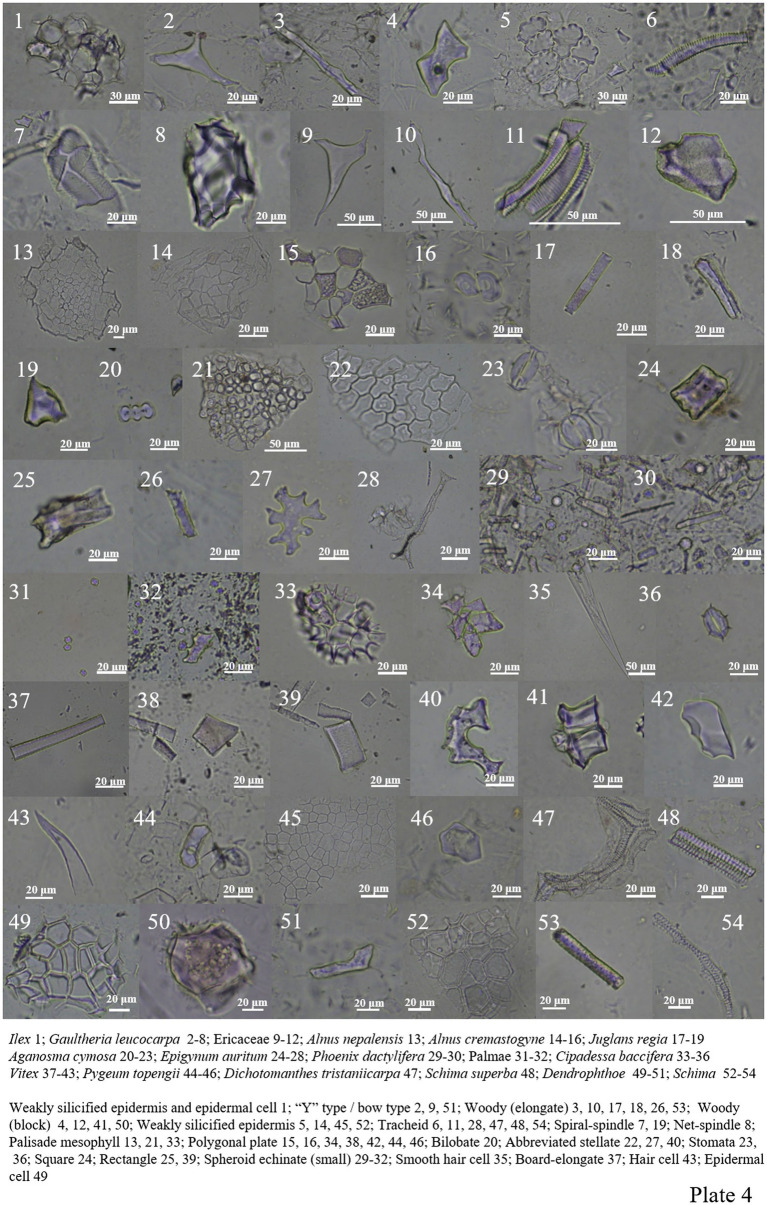


Only *Ilex* was analyzed in Aquifoliaceae, which contained fewer phytoliths and only weakly silicified epidermis and epidermal cell (Plate 4, 1).

Ericaceae contains a large number of phytoliths, mainly including woody-elongate, woody-block, “Y” type/bow type, tracheid, weakly silicified epidermis, and numerous spiral-spindles and net-spindles (Plate 4, 2–12).

The phytolith content in Betulaceae is relatively low, and only polygonal plate, palisade mesophyll and weakly silicide epidermis (Plate 4, 13–16).

*Juglans regia* was analyzed in Juglandaceae, which contains two forms, woody-elongate and spiral-spindle, among which spiral-spindle has a large content (Plate 4, 17–19).

Apocynaceae, including square, rectangle, abbreviated stellate, and palisade mesophyll, etc. (Plate 4, 20–28).

Palme plants contain abundant phytoliths (Xu D. K. et al., 2005; Fenwick et al., 2011), *Phoenix dactylifera* and one unknown species mainly included two types of phytoliths: spheroid echinate (small, <20 μm) and thorn-elongate (Plate 4, 29–32).

Four phytoliths have been found in the Meliaceae plant *Cipadessa baccifera*, including palisade mesophyll, polygonal plate, smooth hair cell, and siliconized stomata (Plate 4, 33–36).

*Vitex* common phytolith types include abbreviated stellate (there are many), polygonal plate, plate-elongate, rectangle, woody-block, and hair cell (Plate 4, 37–43).

*Pygeum Topengii* contains a large number of abbreviated stellate epidermal cells, and *Dichotomanthes tristaniicarpa* contains a large number of tracheids (Plate 4, 44–47).

The *Dendrophthoe* of Loranthaceae mainly includes epidermal cell, woody-block, and “Y” type/bow type (Plate 4, 49–51).

**PLATE 5:**
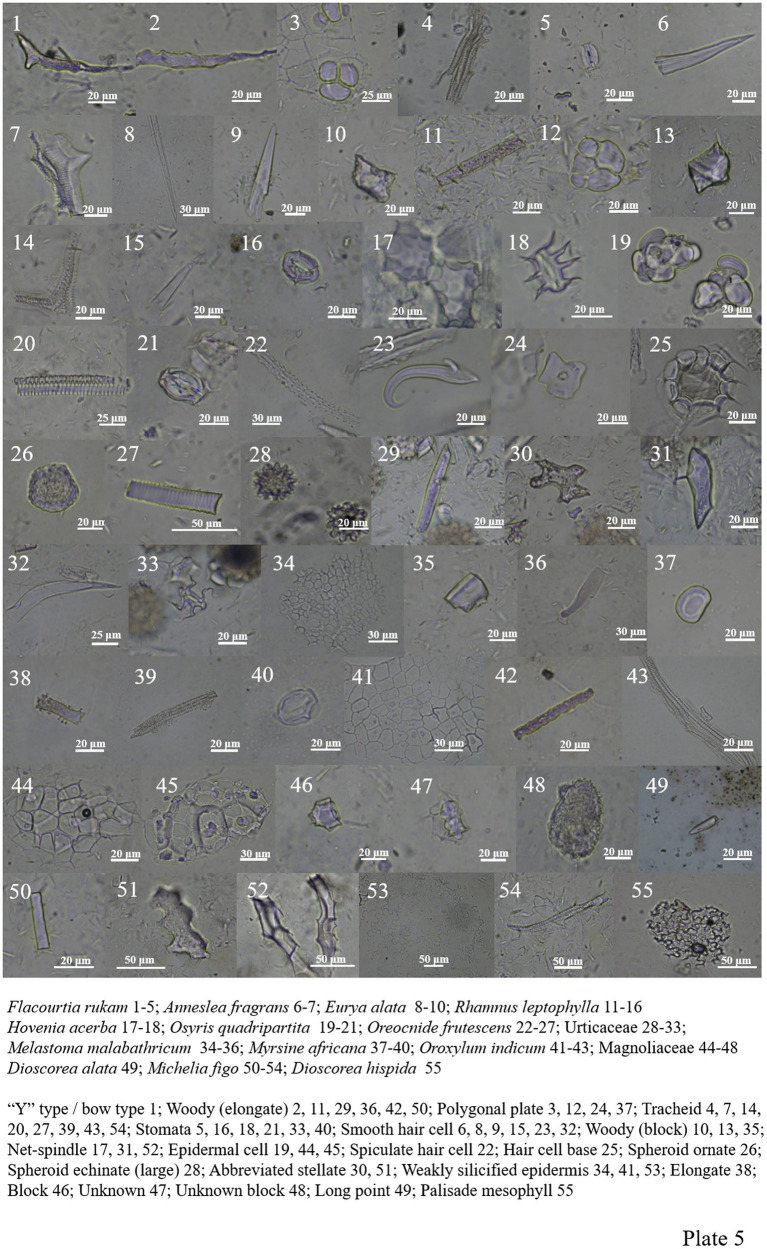


*Flacourtia rukam* of Salicaceae includes the “Y” type/bow type, woody-elongate, polygonal plate, tracheid, and siliconized stomata (Plate 5, 1–5).

A small amount of phytoliths is found in Theaceae plants. In *Schima*, there are mainly weakly silicified epidermis, wood elongates and tracheids. *Anneslea fragrans* contains smooth hair cell and tracheid. *Eurya alata* contains smooth hair cells and woody blocks; phytoliths are not included in *Camellia sinensis* (Plate 4, 48, 52–54; Plate 5, 6–10).

Rhamnaceae contains woody-elongate, woody-block, tracheid, polygonal plate, smooth hair cell, net-spindle, siliconized stomata and silicified hair base (Plate 5, 11–18).

*Osyris quadripartite* of Santalaceae contains three types of phytoliths: tracheid, siliconized stomata, and numerous epidermal cells (Plate 5, 19–21).

Only one block is found in *Trevesia palmata* of Araliaceae.

Phytolith is abundant in Urticaceae. In *Oreocnide frutescens*, the content of spiculate hair cell and smooth hair cell is relatively higher, and other forms include polygonal plate, spheroid ornate, tracheid and hair base. The other Urticaceae plant contains more forms include spheroid echinate (large), woody-elongate, abbreviated stellate, net-spindle, smooth hair cell, siliconized stomata, and its seeds also contain a large number of smooth hair cells (Plate 5, 22–33).

*Melastoma Malabathricum* of Melastomataceae has a low phytolith content, with only a weakly silicified epidermis, woody-block, and woody-elongate (Plate 5, 34–36).

*Myrsine africana* includes four types of phytoliths: polygonal plate, elongate, tracheid, and siliconized stomata (Plate 5, 37–40).

*Oroxylum indicum* of Bignoniaceae includes weakly silicified epidermis, woody-elongate, and tracheid (Plate 5, 41–43).

The unknown species of Magnoliaceae include epidermal cell, block, and unknown block, and the *Michelia figo* includes woody-elongate, abbreviated stellate, net-spindle, tracheid, and weakly silicified epidermis (Plate 5, 44–48; 50–54).

**PLATE 6:**
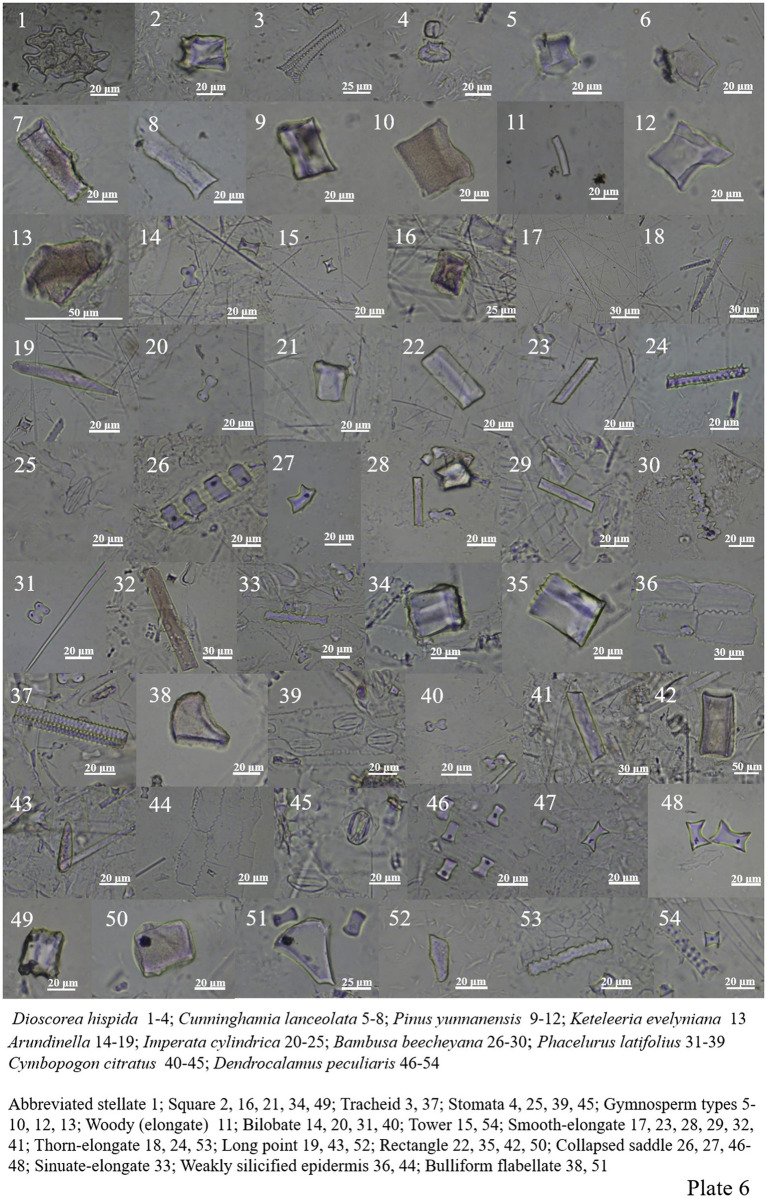


Dioscoreaceae includes abbreviated stellate, tracheid, square, palisade mesophyll, siliconized stomata, and long point (Plate 5, 49, 55; Plate 6, 1–4).

*Cunninghamia lanceolata* in Cupressaceae is rich in phytolith, including cube, woody-elongate and stone-like. *Pinus yunnanensis* in Pinaceae contains three types of phytoliths, including woody-elongate, cube and stone-like, and *Keteleeria evelyniana* only one stone-like (Plate 6, 5–13).

(2) Phytolith morphology of herbaceous plants.

**PLATE 7:**
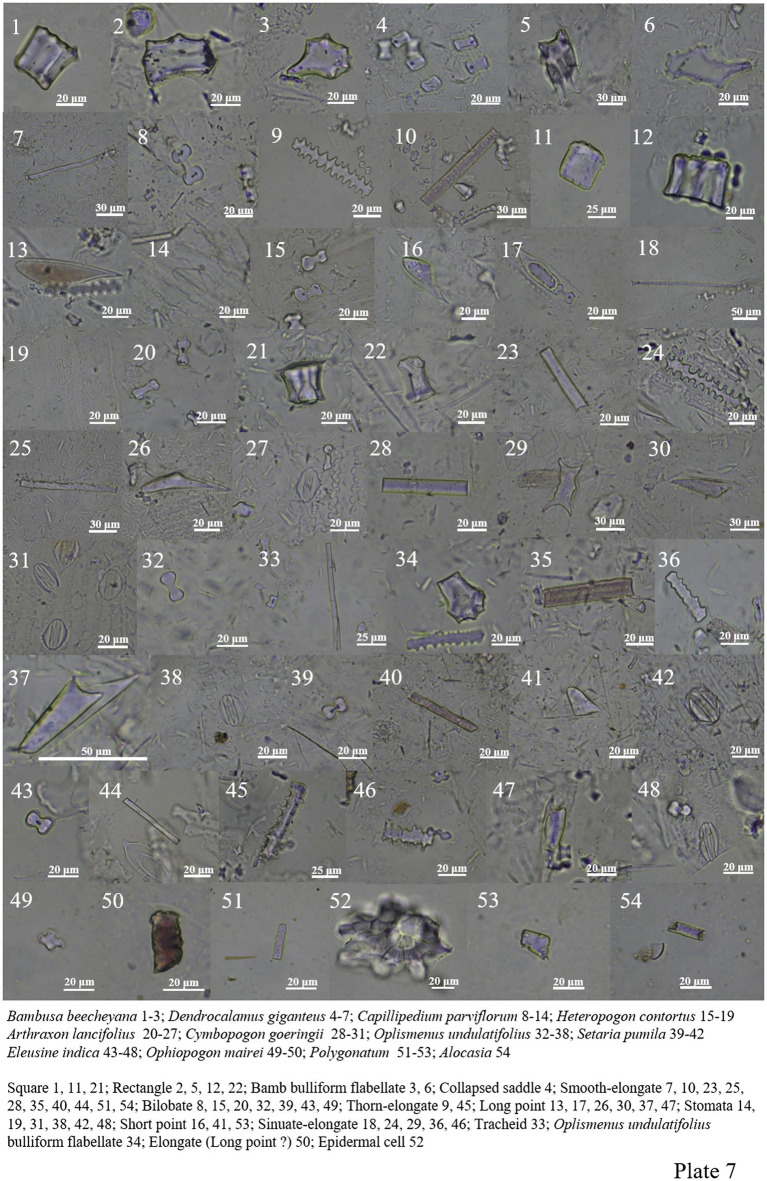


Poaceae plants are widely distributed in different environments. Bilobate, smooth-elongate, thorn-elongate, sinuate-elongate, square, rectangle, long point and short point all appear in the samples. Collapsed saddle and bamb bulliform flabellate are characteristic of *Dendrocalamus giganteus* and *Dendrocalamus peculiaris* (Plate 6, 14–54; Plate 7, 1–48).

*Ophiopogon Mairei* contained less phytolith in Asparagaceae, and only bilobate and elongate were found (Plate 7, 49–50). Phytolith forms of *Polygonatum* are smooth-elongate, epidermal cell, and short point (Plate 7, 51–53).

**PLATE 8:**
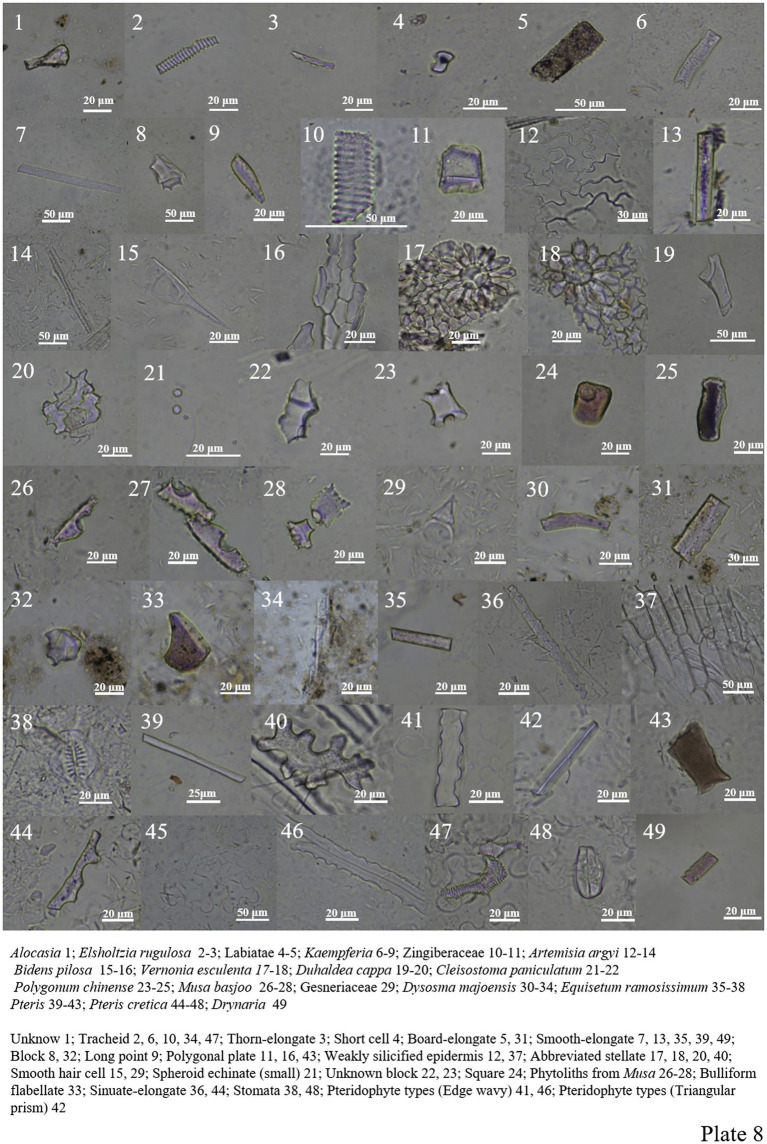


Only smooth-elongate and unknown block phytoliths are seen in *Alocasia* (Plate 7, 54; Plate 8, 1).

The phytolith content in Labiatae is very low, with only a small amount of plate-elongate, tracheid types, etc. (Plate 8, 2–5).

The phytolith forms of Zingiberaceae include smooth-elongate, tracheid, polygonal plate, and long point types (Plate 8, 6–11).

Asteraceae has analyzed phytolith morphology in four plants, all of which have a high number of Epidermal cells. The tracheid, smooth-elongate types, are found in *Artemisia Argyi*. Smooth hair cells are found in *Bidens pilosa* (Plate 8, 12–20).

*Polygonum chinense* contains fewer phytolith, and only three different types are found in the roots, including unknown block, square, and smooth-elongate types (Plate 8, 23–25).

*Musa Basjoo* in Musaceae contains phytoliths that can be identified, including two cavate forms. Of course, a previous study by Premathilake et al. (2018) also compared these two forms in some detail (Plate 8, 26–28).

Gesneriaceae contains a small amount of phytolith and only one hair cell (Plate 8, 29).

*Cleisostoma paniculatum* in Orchidaceae contains a large number of spheroid echinate phytoliths with small individual forms (Plate 8, 21–22).

*Dysosma majoensis* in Berberidaceae contains smooth-elongate, plate-elongate, bulliform flabellate, tracheid and block types (Plate 8, 30–34).

*Equisetum ramosissimum* in Equisetaceae contains smooth-elongate, sinuate-elongate, weakly silicified epidermis and siliconized stomata types (Plate 8, 35–38).

(3) Phytolith morphology of pteridophytes.

**PLATE 9:**
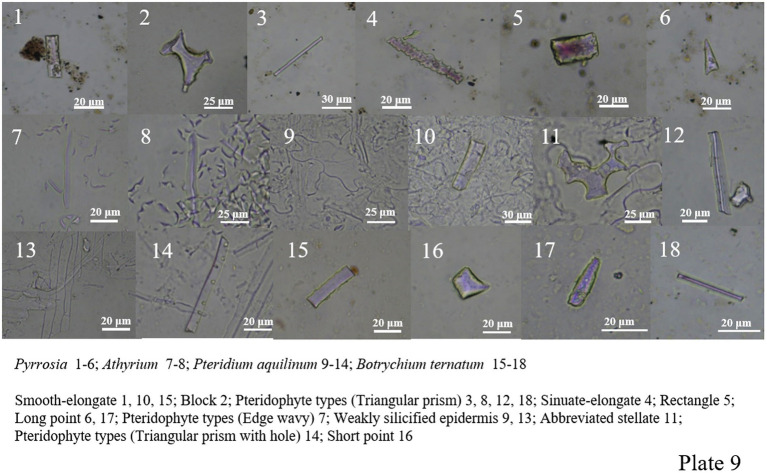


*Pteris*, *Pteris cretica*, *Drynaria*, *Pyrrosia*, *Athyrium*, *Botrychium ternatum*, *Pteridium aquilinum* and so on were analyzed. Ferns produced many different phytoliths morphologies, including smooth-elongate, sinuate-elongate, block, abbreviated stellate, polygonal plate, wavy edge, triangular prism, triangular prism with scrobiculate, tracheid, rectangle, long point, short point, and siliconized stomata. Among ferns, the morphological types with distinguishing characteristics are mainly wavy and triangular prisms and triangular prisms with scrobiculate (Plate 8, 39–49; Plate 9, 1–18).

### Phytolith assemblages in surface soil from the research region

**Figure 3:**
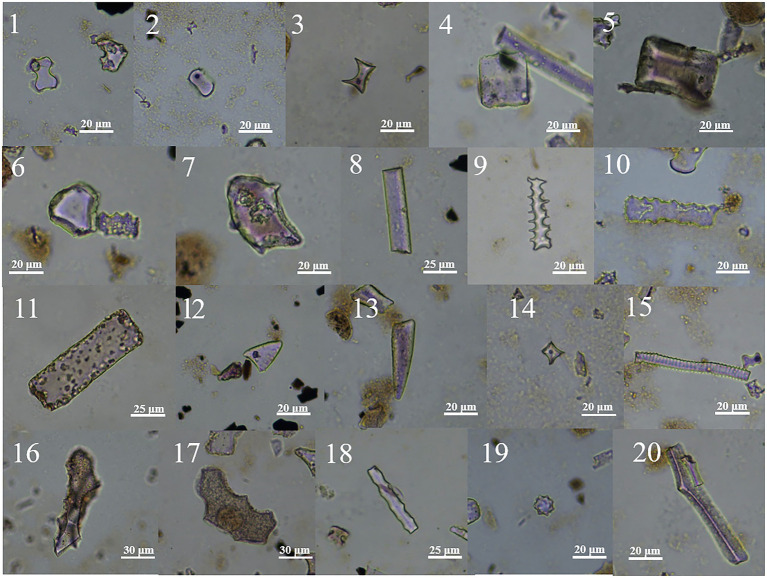
Diagnosis morphotypes of phytoliths in surface soil 1. Bilobate 2, 3. Collapsed saddle 4. Square 5. Rectangle 6. Bulliform flabellate 7. Bamb bulliform flabellate 8. Smooth-elongate 9. Thorn-elongate 10. Sinuate-elongate 11. Board-elongate 12. Short point 13. Long point 14. Tower 15. Tracheid 16. Net- spindle 17. Abbreviated stellate 18. Woody-elongate 19. Spheroid echinate (small) 20. Pteridophyte type.

Phytoliths in 19 surface soil samples were analyzed, and a total of 12,140 grains were tallied and identified. There are 35 diagnostic morphotypes, mainly including bilobate, collapsed saddle, square, rectangle, cuneiform, bamb bulliform flabellate, thorn-elongate, sinuate-elongate, plate-elongate, short point, long point, tower, tracheid, net-spindle, abbreviated stellate, woody-elongate, globular echinate, and triangular prism (Figure 3). However, some morphotypes identified in plant samples were not found in surface sample assemblages, including hair cell, hair cell base, palisade mesophyll, siliconized stomata, and cavate of *Musa basjoo*.

**Figure 4:**
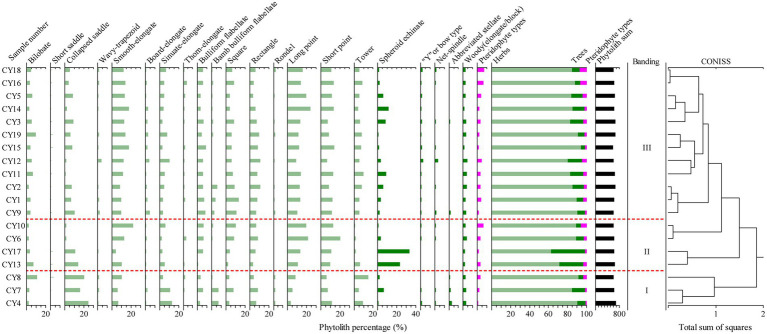
Chart of the percentage content of topsoil phytoliths in the research area.

The phytolith assemblages of surface soil can be divided into three zones, Ph-I, Ph-II and Ph-III, according to a cluster analysis by Tilia (Figure 4), as follows:

Ph-I: The main phytolith assemblage of Ph-I is the collapsed saddle-bamb bulliform flabellate -square/rectangle. This zone includes three sample sites (CY4, CY7 and CY8) from a bamboo-broad-leaved forest in a warm lowland with altitudes ranging from 1,000 ~ 1,300 m. The main vegetation communities are *Pinus*, natural bamboo forest and wild bamboo forest, and beneath there are Poaceae and *Ageratina adenophora*. In this phytolith assemblage, collapsed saddle accounts for 20.2% and has the highest content, followed by sinuate-elongate (9%), short point (8.1%), long point (5.3%), bamb bulliform flabellate (4.7%), square (4.7%), and rectangle (6.1%). Among the three sample sites, CY7 has the most spheroid echinate (small), accounting for 6.2%. The collapsed saddle and bamb bulliform flabellate make up the largest portions of samples collected in the bamboo forest area.

Ph-II: The main phytolith assemblage is spheroid echinate (small)-woody (elongate/block). This zone includes four sample sites (CY6, CY10, CY13 and CY17) collected from warm broad-leaved forest and warm coniferous forest (containing an appreciable quantity of Palmae) with altitudes ranging from 1,300 ~ 1,950 m. The main vegetation communities are *Quercus,* Euphorbiaceae, Fabaceae, Betulaceae, Acanthaceae, etc., a*nd* beneath there are *Phoenix loureiroi*, Poaceae, *Duhaldea cappa*, *Ageratina adenophora* and Pteridophyta. In this phytolith assemblage, the content of spheroid echinate (small) was the highest, ranging from 0.3 to 33.4%, with an average content of 14.9%. The average content of woody (elongate/block) is 2.2%. The main diagnostic phytolith morphotype of surface soil samples collected in the Palmae *area* is spheroid echinate (Palmae type).

Ph -III: The main phytolith assemblage is elongate-point-spheroid echinate (small). This zone includes 12 sampling sites (CY1, CY2, CY3, CY5, CY9, CY11, CY12, CY14, CY15, CY16, CY18 and CY19) main collected from warm broad-leaved forest and warm coniferous forest with altitudes ranging from 1,300 ~ 2,100 m. The main vegetation communities are *Quercus*, Betulaceae, Fabaceae, Moraceae, Theaceae, Lauraceae and *Pinus*. And beneath there are Poaceae, *Ageratina adenophora*, *Artemisia*. In this phytolith assemblage, Poaceae accounts for a large proportion. The main morphotypes are long point (13.8%), short point (10.7%) and smooth-elongate (12.4%), followed by rectangle (7.3%), square (7.2%), Bulliform flabellate (5.6%), and sinuate-elongate (4.9%). Spheroid echinate (small) accounts for 4.1%, “Y” type/bow type is 1.1%, and woody (elongate/block) type accounts for 2.6%.

### Pollen assemblages in surface soil from the Cangyuan region

**Figure 5:**
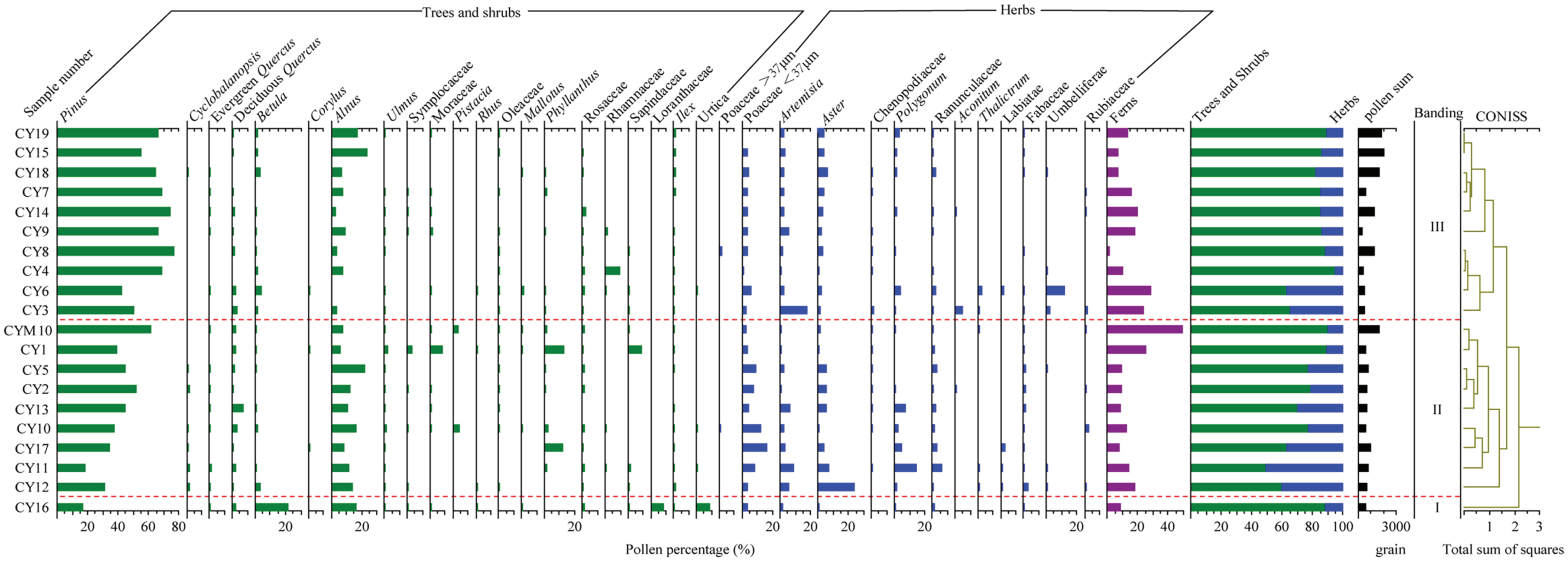
Chart of the percentage of topsoil pollen in the research area.

Pollens in 19 surface soil samples and one moss sample were analyzed. The identified pollens belong to 97 families and 79 genera (49 families). Most of the subtropical semihumid evergreen broad-leaved forest pollen types are included in the assemblages, which are dominated by *Pinus* (average content 50.5%), *Alnus* (10.2%), Poaceae (<37 μm; 5%), *Artemisia* (3.6%), *Aster* (4.1%), etc. Pollen assemblages of surface soil can be divided into three zones, P-I, P-II and P-III, according to a cluster analysis by Tilia (Figure 5).

P-I: Pollen assemblages represented by *Betula-Pinus-Alnus* have only one sampling site, CY16. The site is located in evergreen and deciduous broad-leaved forest, with an altitude of 1,839 m. The main vegetation communities are *Betula and* Theaceae *schima*. Arboreal plants dominated in the pollen assemblages, including *Pinus* (16.7%), *Betula* (21.8%), *Alnus* (14%), and Loranthaceae (8.4%). Shrub (Semishrub) plants are mainly Urticaceae (8.8%). The content of herbaceous plants, including Poaceae (< 37 μm; 3.6%) and *Artemisia* (1.2%), is relatively low. Very few ferns are found in the assemblage. The vegetation in the sampling site is mainly deciduous Betulaceae trees, and the pollen assemblage can reflect the vegetation community well.

P-II: The pollen assemblage mainly includes *Pinus*-*Alnus*-Poaceae-*Artemisia.* This zone has 8 surface sampling sites (with altitudes ranging from 1,050–1,950 m) and 1 moss sample (CYM10). *Quercus*, Fabaceae, Moraceae, *Eurya japonica*, etc., are dominant plants in those sampling sites, which are warm broad-leaved forest belts. Poaceae, *Artemisia* and other herbs grow under the forest. The pollen assemblages mainly include *Pinus* (40.2%), *Alnus* (11.9%), and deciduous *Quercus* (2.2%). Pollens from herbaceous plants showed obvious changes, including Poaceae (<37 μm; 7.4%), *Artemisia* (3.4%), *Aster* (5.8%), and *Polygonum* (3.5%). The pollen assemblages indicate a relatively high content of *Pinus* and *Alnus*, while those of modern vegetation *Pinus* and *Alnus* are less abundant, which shows that their pollen assemblage is overrepresented. However, Fabaceae and Fagaceae pollen have low representativeness. Mosses abound in CY10 and CYM10, and pollen assemblages also indicate the same situation. Surface soil sampled in CYM10 had the highest fern pollen content, followed by CY1.

P-III: The pollen assemblage is dominated by *Pinus,* followed by *Alnus*. This zone has 10 surface sampling sites (with altitudes ranging from 1,050–2,100 m). The main vegetation communities are Fagaceae, *Pinus*, Fabaceae, Acanthaceae, Theaceae, *Alnus,* etc.*, a*nd under there are Poaceae and *Ageratina adenophora*. Arboreal plants dominate in this pollen assemblage. Compared to P-I and P-II, *Pinus* had a higher content (63.2%), followed by *Alnus* (8.1%). Herbaceous plant pollen has a relatively low content, mainly including Poaceae (<37 μm; 3%), *Artemisia* (4%), *Aster* (2.8%)*, and Polygonum* (1.3%). Apiaceae accounted for 11.4% in CY6. The content of pteridophytes was slightly lower than that of P-II. Pollen assemblages of sampling sites containing a high content of *Pinus*, *Alnus*, and Poaceae are consistent with modern vegetation.

## Discussion

### Significance of topsoil phytolith and sporo-pollen assemblages for modern vegetation communities

In this study, we probe the relationship between phytolith and sporo-pollen assemblages as well as the surrounding vegetation in the subtropical semihumid broad-leaved evergreen forest of Hengduan Mountain, western Yunnan. We attempt to determine the phytolith and sporo-pollen assemblages that can indicate the subtropical semihumid broad-leaved evergreen forest in this region to provide basic information for the reconstruction of the paleovegetation.

The AP/NAP value in this area was greater than 1, indicating that this area was a forest area (tree pollen content >50%, herb pollen content <45%). The pollen and phytolith in this area were analyzed by PCA and compared with altitude. The results showed that the correlation was weak (The correlation between pollen PC1 + PC2 and altitude was *R*^2^ = 0.048, and the correlation between phytolith PC1 + PC2 and altitude was *R*^2^ = 0.05), which had little relationship with altitude. But the surface soil phytoliths have good correspondence with sampling site vegetation, indicating that phytoliths have obvious characteristics of *in situ* deposition. In subtropical lowland and mountainous evergreen broad-leaved forests in the study area, constructive species mainly come from Fagaceae and Fabaceae. In this study, 9 species of Fagaceae and 9 species of Fabaceae modern sample plants were mainly produced, including “Y” type/bow type, spiral-spindle, woody (elongate/block), tracheid, abbreviated stellate, polygonal plate, globular smooth, palisade mesophyll, smooth hair cell, and siliconized stomata. In the warm broad-leaved forest, warm coniferous forest with topsoil phytolith combination contain a small amount of “Y” type/bow type and spiral-spindle, but the proportion of woody plants is relatively small, and the gramineous type phytolith content has a higher percentage. In the bamboo forest area, the contents of the collapsed saddle and bamb bulliform flabellate are higher. Topsoil samples containing Palmae plants are dominated by spheroid echinate (small). This shows that a small range of actual building species can also be reflected in topsoil phytoliths. Phytolith assemblages of topsoil plants in the study area may partly reflect constructive species (e.g., Bambusoideae, Palmae), but most of the samples reflected some nonestablishment species (e.g., Poaceae overrepresented).

The surface soil spore-pollen combination can better reflect the vegetation type in the study area, and the indication of vegetation at the sampling point is slightly weaker than that of phytoliths. Surface soil pollen assemblages with a high pollen content of *Pinus* show overrepresented characteristics. Most of the sampling sites (CY1, CY2) were Fabaceae and Moraceae, and the pollen of these two plants accounted for a higher proportion in the pollen assemblage of topsoil. The pollen assemblage of Betulaceae (CY3, CY16, CY18) was dominated by *Alnus*, reaching 16% at CY16, indicating that pollen has good indication for local vegetation. However, the pollen contents of *Quercus* in the pollen assemblages of the samples (CY5, CY9, CY10, CY13, CY15) were generally low, with a high value (7.3%) only at CY13, indicating that the pollen of *Quercus* was weakly representative. The above results show that the relationship between surface soil pollen and modern vegetation presents a certain degree of correspondence, but not completely corresponding characteristics, which is related to the long flight distance and widespread of pollen. Therefore, pollen assemblages can reflect the regional (nonlocal) vegetation landscape.

The surface soil phytoliths in the study area are mainly elongate-bulliform flabellate-square/rectangle-woody (including spheroid echinate (small)). Compared with temperate, subtropical and tropical arid, semiarid phytoliths (Zhang et al., 2008; An and Lu, 2010; Bai et al., 2020), this combination has a unique representation of subtropical low latitude mountain vegetation zones. Topsoil pollen assemblages are dominated by *Pinus yunnanensis*-*Betula*-deciduous *Quercus*-Euphorbiaceae-Rhamnaceae and have typical subtropical low latitude mountain vegetation characteristics in southwest China.

### Indicative significance of surface soil phytoliths and spore-pollen assemblages to climate and environment

Different combinations of topsoil can indicate different vegetation features, thus reflecting local climatic conditions. For example, spherical phytoliths are common in topsoil phytoliths in tropical regions (Barboni and Bremond, 2007; Dickau et al., 2009). In the subtropics of China, Gongga Mountain is dominated by a “Y” type/bow type-bilobate-cruciform-saddle combination (An and Lu, 2010). The phytolith assemblages in our study area are mainly the elongate-bulliform flabellate-square/rectangle-wood (including spheroid echinate) assemblages, and the indicator of the phytolith assemblage type is more inclined to subtropical climate. In particular, the vegetation belt dominated by spheroid echinate (small) phytoliths reflects that the vegetation combination types include Palmae plants growing in tropical and subtropical regions.

In the subtropical forests of the study area, there are many Poaceae plants and abundant phytoliths, so they have good performance in the combination of topsoil phytoliths. In general, the proportion of Poaceae phytoliths in the coniferous and broad-leaved mixed forest belt is large, and it is more likely to be overrepresented in warm coniferous forest and warm broad-leaved forest belts. In this study, the phytolith assemblages of Poaceae showed obvious regularity. With the change in altitude from high to low, the percentages of the collapsed saddle and square/rectangle type increased. The long point type showed a decreasing trend with decreasing altitude. Poaceae plants represented by bilobate, square, rectangle, and tower morphologies are mostly found in the middle of the mountain (approximately 1,300–2,000 m). For a long time, the study of Poaceae plants has been relatively mature, and the combined morphology of Poaceae plants can also indicate basic local environmental characteristics. In general, long point phytoliths are dry and cold, collapsed saddle phytoliths are wet, and bulliform flabellate phytoliths are warm (Ge, 2016). In general, the phytolith assemblages of Poaceae in this area were dominated by collapsed saddle-bulliform flabellate-square/rectangle-elongate-point assemblages, reflecting the warm and humid environmental conditions in this area. Combined with the characteristics of the woody phytolith assemblage, it can be determined that the climate is warm and humid, which is in good agreement with the local climate that belongs to the subtropical low latitude mountain monsoon climate. Therefore, the use of phytolith assemblages can better indicate the climatic conditions of the study area, thus providing a basis for paleoclimate research.

The pollen assemblage characteristics can also reflect the vegetation types in different habitats, indicating the climate environment represented by them. The main pollen types in this area are *Pinus yunnanensis*, deciduous *Quercus*, *Betula*, *Alnus*, Moraceae, Rhamnaceae and *Polygonum*. *Pinus yunnanensis* is mainly distributed in the region of 13–18°C (23°–29° N, 98°30′–105° E; Li and Liu, 1984). Deciduous *Quercus* is widely distributed in subtropical evergreen broad-leaved forests, with southwestern China as the distribution center (Wang et al., 1985). *Betula* and *Alnus* are distributed throughout China and have a wide range of habitats. Moraceae is most common south of the Yangtze River (Anhui Flora Cooperative Group, 1987). There are approximately 600 species of Euphorbiaceae, which are widely distributed in tropical and subtropical regions (Guan et al., 2004). The richness of genera and species in the southern region of Rhamnaceae was significantly higher than that in the northern region; that is, the spatial distribution diversity pattern was higher in the south and lower in the north (Li et al., 2021). Yunnan Province is the distribution center of richness of species and genus of Rhamnaceae in China (Huang, 2014). Polygonum is mainly distributed in the Hengduan Mountains, extending westward along the Himalayan Mountains (Zhao and Hou, 2011). In summary, pollen assemblages reflect that the region is mainly a tropical-subtropical plant community, indicating warm and humid climatic conditions.

## Conclusion

After the analysis of modern plant samples and topsoil pollen and phytolith assemblages in the semihumid evergreen broad-leaved forest region of Hengduan Mountain in western Yunnan, the following conclusions are drawn: There is a good correspondence between the morphological types of phytoliths in surface soil and modern plants in subtropical low latitude mountainous areas. The surface soil phytoliths and vegetation in this area have good correspondence, and the small-scale actual constructive species can also be reflected in the surface soil phytoliths. In the samples collected from the bamboo forest area and Palmae plants, the percentages of collapsed saddle, bamb bulliform flabellate and spheroid echinate (small) were higher. The pollen samples of topsoil corresponded to the species of modern vegetation surveyed in this area, but *Pinus* was overrepresented, and *Quercus* was poorly representative. Pollen and phytolith assemblages have unique combination characteristics, with a strong low latitude subtropical monsoon climate representative.

## Data availability statement

The original contributions presented in the study are included in the article/supplementary material, further inquiries can be directed to the corresponding author.

## Author contributions

All authors listed have made a substantial, direct, and intellectual contribution to the work and approved it for publication.

## Funding

This work was jointly supported by the National Natural Science Foundation of China (41991323, U1902208, 41672344), the Strategic Priority Research Program of Chinese Academy of Sciences (XDB26020301), the Second Tibetan Plateau Scientifc Expedition and Research (STEP) (2019QZKK0704), and Yunnan Leading Talent Project (202005AB160008).

## Conflict of interest

The authors declare that the research was conducted in the absence of any commercial or financial relationships that could be construed as a potential conflict of interest.

## Publisher’s note

All claims expressed in this article are solely those of the authors and do not necessarily represent those of their affiliated organizations, or those of the publisher, the editors and the reviewers. Any product that may be evaluated in this article, or claim that may be made by its manufacturer, is not guaranteed or endorsed by the publisher.

## Footnotes

^1^http://data.cma.cn/ (Accessed August 26, 2022).
